# The phytochemical and pharmacological profile of taraxasterol

**DOI:** 10.3389/fphar.2022.927365

**Published:** 2022-08-04

**Authors:** Fengjuan Jiao, Zengyue Tan, Zhonghua Yu, Bojie Zhou, Lingyan Meng, Xinyue Shi

**Affiliations:** ^1^ Shandong Collaborative Innovation Center for Diagnosis, Treatment and Behavioral Interventions of Mental Disorders, Institute of Mental Health, Jining Medical University, Jining, China; ^2^ Shandong Key Laboratory of Behavioral Medicine, School of Mental Health, Jining Medical University, Jining, China

**Keywords:** taraxasterol, dandelion, botany, pharmacological profile, phytochemistry

## Abstract

Taraxasterol is one of the bioactive triterpenoids found in dandelion, a member of the family Asteraceae. In the animal or cellular models of several ailments, including liver damage, gastritis, colitis, arthritis, pneumonia, tumors, and immune system diseases, taraxasterol has been shown to have significant preventive and therapeutic effects. This review aims to evaluate the current state of research and provide an overview of the possible applications of taraxasterol in various diseases. The reported phytochemical properties and pharmacological actions of taraxasterol, including anti-inflammatory, anti-oxidative, and anti-carcinogenic properties, and its potential molecular mechanisms in developing these diseases are highlighted. Finally, we further explored whether taraxasterol has protective effects on neuronal death in neurodegenerative diseases. In addition, more animal and clinical studies are also required on the metabolism, bioavailability, and safety of taraxasterol to support its applications in pharmaceuticals and medicine.

## Introduction

Dandelion is a member of the family Asteraceae and is widely distributed in the warmer temperate zones of the Northern Hemisphere (Gonzalez-Castejon et al., 2012). The plant dandelion has long been used as a medicinal herb. Its therapeutic role was mentioned as early as the 10th and 11th centuries by Arabian physicians for the treatment of liver and spleen diseases (Faber, 1958). In traditional Chinese medicine, dandelion is used in combination with other herbs to treat hepatitis and enhance the immune response to upper respiratory tract infections, bronchitis, and pneumonia (Sweeney et al., 2005).

Medicinal plants typically contain several different chemical compounds that may act individually or synergistically to improve health (Gurib-Fakim, 2006). As one of the bioactive triterpenoids found in dandelion, taraxasterol has become a focus of pharmacological studies. Power and Browning were the first to report the isolation of taraxasterol from the non-saponifiable matter of *Taraxacum officinale* root (Power and Browning, 1912). They first described taraxasterol as a phytosterol. Recently, taraxasterol has received increased attention for its anti-inflammatory, anti-oxidative, and anti-carcinogenic activity and its possible beneficial effects against the development of liver damage, cancer, and numerous immune system diseases. This review aims to evaluate the properties of taraxasterol and investigate its phytochemical properties, focusing on the most recent literature analyzing the pharmacological effects of taraxasterol on several diseases.

## Phytochemical properties of taraxasterol

**FIGURE 1:**
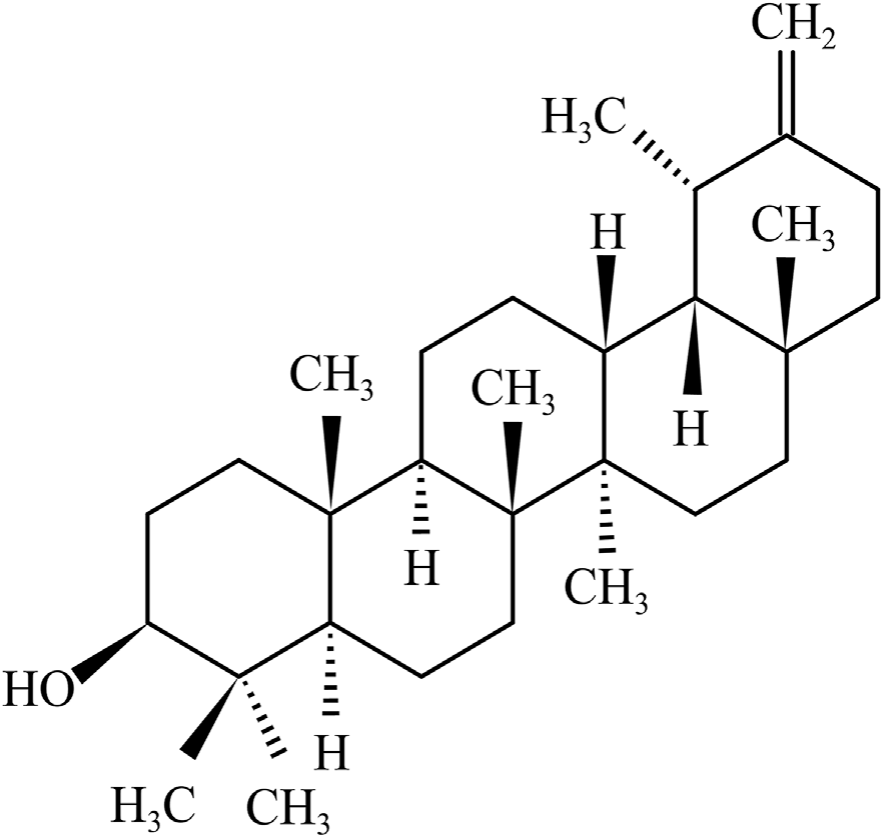
hemical structure of taraxasterol.

Taraxasterol, also known as (3β, 18α, 19α)-Urs-20 (30)-en-3-ol, is a pentacyclic triterpene with a 1,2-cyclopentene phenanthrene structure. The molecular formula of taraxasterol is C_30_H_50_O, and its molecular weight and melting point are 426.72 g/mol and 221–222°C, respectively. In the 1950s, the structure and configuration of taraxasterol were reported by **(** Figure 1) Ames et al. (1954). Oxidosqualene cyclases (OSCs) catalyzed 2,3-oxidosqualene cyclization, which produced triterpene scaffolds (Thimmappa et al., 2014). Recently, it was found that transgenic yeast expressing LsOSC1, one putative lettuce OSC gene, can produce taraxasterol in lettuce (*Lactuca sativa*) (Choi et al., 2020).

Power and Browning discovered taraxasterol and first reported it from the non-saponifiable matter of *Taraxacum officinale* or Wiggers root (Power and Browning, 1912). The highest levels of taraxasterol were observed in the latex of *Taraxacum officinale* (Burrows and Simpson, 1938; Furuno et al., 1993; Akashi et al., 1994). Taraxasterol was also isolated from wild plants and regenerated organs of *Taraxacum officinale* using a reversed-phase HPLC with CH_3_CN/H_2_O (Furuno et al., 1993). In addition, Akash et al. found that the radioactivity of taraxasterol was mainly observed in differentiated organs of *Taraxacum officinale* with accumulation patterns by HPLC combined with liquid scintillation analysis (Akashi et al., 1994). In this study, the biosynthesis of taraxasterol was revealed by detecting the incorporation time course of radioactivity from [2–14 C] mevalonic acid into individual triterpenols in the shoot segments. It was discovered that taraxasterol was synthesized during the first 24 h of the experiment, with the (pseudo)laticifer cells being the probable site of its biosynthesis (Akashi et al., 1994). According to the HPLC analysis, Sharma et al. reported that the quantity of taraxasterol in the natural root extract of *T. officinale* was 2.96 μg/ml, whereas the quantity of taraxasterol was 3.013 μg/ml in the root callus cultures (Sharma and Zafar, 2014). However, the absolute quantitation of taraxasterol in any plant material is unavailable and needs to be further studied. In addition, taraxasterol is present in esculent plants such as legumes, cereals, nuts, and seeds and in plant oils (Xu et al., 2004). Taraxasterol is obtained from various medicinal plants in addition to esculent ones. The distribution of taraxasterol in plants is summarized in Table 1.

**TABLE 1:** edicinal plants containing taraxasterol.

Name of plant	Part containing taraxasterol	References
*Taraxacum officinale*, Wiggers (Asteraceae)	Roots	(Power and Browning, 1912; Burrows and Simpson, 1938)
*Taraxacum officinale* Webers (Asteraceae)	Roots	(Bajaj, 1994; Della Loggia et al., 1994)
*Calendula officinalis* Hohen. (Asteraceae)	Flowers	Akihisa et al. (1996)
*Taraxacum japonicum* Koidz. (Asteraceae)	Roots	Takasaki et al. (1999)
*Hemistepta lyrata* (Bunge) Bunge (Asteraceae)	Whole plant	Ren and Yang (2001)
*Carthamus lanatus* Linn. (Asteraceae)	Aerial parts	Ganeva et al. (2003)
*Taraxacum platycarpum* Dahlst. (Asteraceae)	Roots	Ryu and Lee (2006)
*Mikania cordifolia* (L.f.) Willd. (Asteraceae)	Aerial parts	Oliveira et al. (2006)
*Hieracium pilosella* L. (Asteraceae)	Rhizomes	Gawronska-Grzywacz and Krzaczek (2007)
*Achillea millefolium* Linn. (Asteraceae)	Leaves	Gudaitytė and Venskutonis (2007)
*Taraxacum mongolicum* Hand.-Mazz. (Asteraceae)	Roots	Yarnell and Abascal (2009)
*Cichorium glandulosum* Boiss. et Huet. (Asteraceae)	Air-dried stems	Wu et al. (2011)
*Centipeda minima* (L.) A. Br. and Asch. (Asteraceae)	Leaves	Ngo and Li (2013)
*Chrysanthemum morifolium* Ramat. (Asteraceae)	Flowers and Aerial parts	(Akihisa et al., 2005; Boutaghane et al., 2013)
*Arctium lappa* L. (Asteraceae)	Aerial parts	Zhao et al. (2014)
*Anthemis mirheydari* Iranshahr (Asteraceae)	Whole plant	Jassbi et al. (2016)
*Arnica* L. (Asteraceae)	Leaves	De Amorim et al. (2016)
*Cyanthillium cinereum* (L.) H. Rob. (Asteraceae)	Whole plants	Thongkhao et al. (2020)
*Cynara cardunculus* L. (Compositae)	Flowers	Yasukawa et al. (2010)
*Camellia japonica* Linn.(Theaceae)	Seed oil	Itoh et al. (1980)
*Acrocarpus fraxinifolius *Wight ex Arn*.* (Fabaceae)	Seed oil	Saeecd et al. (1991)
*Holodiscus discolor* (Pursh) Maxim (Rosaceae)	Leaves	Haladova et al. (2001)
*Strobilanthes callosus* Nees (Acanthaceae)	Aerial parts	Singh et al. (2002)
*Philadelphus coronarius* L. (Hydrangeaceae)	Twigs	Valko et al. (2006)
*Bryophyllum pinnatum* (Lam.) Oken (Crassulaceae)	Aerial parts	Kamboj and Saluja (2009)
*Cornus kousa* F.Buerger ex Hance (Cornaceae)	Fruits	Lee et al. (2010)
*Solanum lycopersicum* L. (Solanaceae)	Fruit and leaves	Wang et al. (2011)
*Euphorbia tirucalli* Linn. (Euphorbiaceae)	Latex and stem	Wang et al. (2011)
*Olea europaea* Linn. (Oleaceae)	Aerial parts	Stiti and Hartmann (2012)
*Ficus carica* L. (Moraceae)	Aerial parts	Chauhan et al. (2012)

## Pharmacological profiles of taraxasterol

**FIGURE 2:**
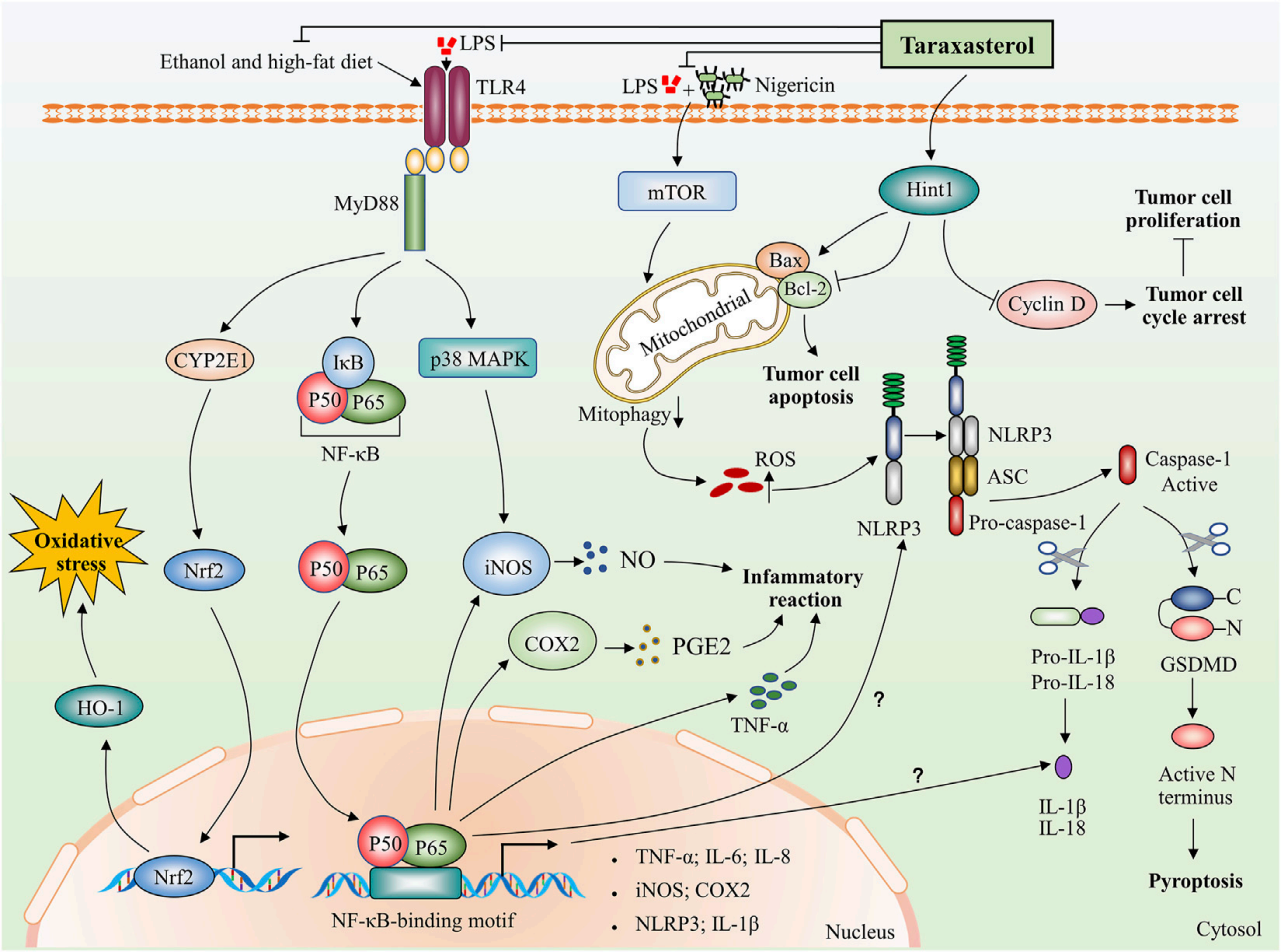
Potential biological mechanisms of taraxasterol: anti-inflammatory, anti-oxidative, and anti-carcinogenic. Taraxasterol impacts several aspects of inflammatory action. On the one hand, taraxasterol reduces the levels of inflammatory cytokines, including TNF-α and IL-6 and reduces serum levels of inflammatory mediators NO and PGE₂ through inhibiting NF-κB and MAPK signaling pathways. On the other hand, taraxasterol reduces ROS-mediated NLRP3 inflammation activation by suppressing mTOR and mitophagy, which contributes to the reduction of the inflammation reaction. Interestingly, NF-κB also regulates the expression of NLRP3 and IL-1β at transcriptional levels, yet the underlying mechanisms of whether the taraxasterol alleviates NLRP3 inflammation activation and reduces the levels of IL-1β by inhibiting the NF-κB signaling pathway are unclear. In addition, taraxasterol reduces oxidative stress induced by ethanol and a high-fat diet by inhibiting the expression of transcription factor Nrf2. Taraxasterol can promote tumor cell apoptosis and inhibit tumor cell proliferation by upregulating Hint1 expression in human liver cancer.

The use of taraxasterol has been linked to many health advantages. The following sections review investigations that support the pharmacological properties ascribed to taraxasterol. The pharmacological activities of taraxasterol in the fight against various diseases *in vitro* and *in vivo* are summarized in Table 2. The mechanism of action of taraxasterol is summarized in Figure 2.

**TABLE 2:** Pharmacological activities of taraxasterol against diseases (*in vitro* and *in vivo* studies)^a^.

Pharmacological activities	Part of plant	Cells and/or animal models of disease	Dose	Mechanisms	References
Anti-inflammatory activity	Aerial parts of *Inula japonica* (Miq.) Komarov	LPS-induced endotoxic shock in mice	2.5, 5 and 10 mg/kg	TNF-α↓, IFN-γ↓, IL-1β↓, IL-6↓, NO↓, and PGE₂↓	Zhang et al. (2014)
	Compositae flowers	TPA-induced inflammation in mice	0.3 mg per ear	Not mentioned	Akihisa et al. (1996)
	Aerial parts of *Inula japonica* (Miq.) Komarov	LPS-induced ALI in mice	10 mg/kg	Inhibition of NF-κB and MAPK pathways	San et al. (2014)
	Aerial parts of *Inula japonica* (Miq.) Komarov	ConA-induced acute hepatic injury in mice	5 and 10 mg/kg	TNF-α↓, IL-6↓, IL-1β↓, IFN-γ↓, and IL-4↓; TLR2↓, TLR4↓, and NF-κB p65↓; Bax/Bc1–2↓	Sang et al. (2019)
	*Taraxacum officinale*	DSS-induced AEC in mice	10 mg/kg	TNF-α↓, IL-1β↓, and IL6↓	Chen et al. (2019b)
	Aerial parts of *Inula japonica* (Miq.) Komarov	HT-29 cells treated with LPS; DSS-induced colitis in mice	Cells 2.5, 5 and 10 μg/ml; animals 25, 50, and 100 mg/kg	IL-6↓, TNF-α↓; p53↓, Bax↓, caspase-3↓	Che et al. (2019)
	Aerial parts of *Inula japonica* (Miq.) Komarov	Primary human chondrocytes treated with IL-1β	2.5, 5, and 10 μg/ml	NO↓, iNOS↓; NF-κB↓; TNF-α↓, IL-6↓, and IL-8↓; NLRP3↓	Piao et al. (2015)
	*Taraxacum mongolicum* Hand-Mazz	Primary HFLS-RA treated with IL-1β; CIA mice	Cells 3, 10, and 30 μM; animals 10 mg/kg	TNF-α↓, IL-6↓, and IL-8↓; NF-κB↓; NLRP3↓	Chen et al. (2019a)
	Aerial parts of *Inula japonica* (Miq.) Komarov	FCA-induced arthritis in rat	2, 4, and 8 mg/kg	TNF-α↓, IL-1β↓, and PGE2↓	Wang et al. (2016)
	Not mentioned	Acne mice	5 and 10 mg/kg	IL-1β↓, IL-8↓, TGF-β1↓, Smad3↓	Liu et al. (2020)
	Aerial parts of *Inula japonica* (Miq.) Komarov	BV2 microglia cells treated with LPS	3, 6, and 12 μg/ml	TNF-α↓, IL-1β↓; NF-κB↓; LXRα↑ and ABCA1↑	Liu et al. (2018)
	Aerial parts of *Inula japonica* (Miq.) Komarov	HUVECs treated with LPS	5,10, and 15 μg/mL	TNF-α↓, IL-8↓, PGE2↓, COX-2 ↓, NF-κB↓, and LXRα↑	Zheng et al. (2018)
Anti-oxidative activity	Aerial parts of *Inula japonica* (Miq.) Komarov	IRI-induced AKI in mice; HK-2 cells stimulated with H/R	Cells 5 and 10 μM; animals 5 and 10 mg/kg	ROS↓, Bax↓, and Bcl2↑	Li et al. (2020a)
	Aerial parts of *Inula japonica* (Miq.) Komarov	Ethanol-induced liver injury in mice	2.5, 5, and 10 mg/kg	ROS↓, MDA↓; GSH↑, and SOD↑	Xu et al. (2018)
	*Taraxacum officinale*	Ethanol and high-fat diet-induced liver injury in mice	2.5, 5, and 10 mg/kg	CYP2E1↓, total and nuclear Nrf2↑, HO-1↑	Li et al. (2020b)
	Aerial parts of *Inula japonica* (Miq.) Komarov	CS-induced lung inflammation in mice	2.5, 5, and 10 mg/kg	Inhibition of TLR4 translocate to lipid rafts; ROS↓	Xueshibojie et al. (2016)
	*Taraxacum mongolicum*	OGD/R-induced hippocampal neurons injury	2.5, 5, and 10 μM	ROS↓, MDA↓, HO-1↑, NQO-1↑, and GPx-3↑; Nuclear Nrf2↑	He et al. (2020)
	Aerial parts of *Inula japonica* (Miq.) Komarov	Cardiomyocyte ischemia/reperfusion mice	5, 10, and 30 μmol/L	SOD↑, MAD↓, p-ERK1/2↑	Wang et al. (2018)
Anti-carcinogenic activity	Tabular flowers of *artichoke*	TPA-induced skin tumor in mice	2.0 μmol	Not mentioned	Yasukawa et al. (1996)
	The herbs of *Taraxacum officinale*	HepG2 and SK-Hep1 cells	17.0 μM	Hint1↑, Bax↑ Bcl2↓, and cyclin D1↓	Bao et al. (2018)
	Aerial parts of *Inula japonica* (Miq.) Komarov	Xenograft tumor model of gastric cancer in mice	25 μg/ml	EGFR↓, total AKT1↓, p-AKT1↓, and p-EGFR↓; RNF31↓, p53↑	Tang et al. (2021)
Anti-allergic activity	Aerial parts of *Inula japonica* (Miq.) Komarov	OVA-induced allergic asthma in mice	2.5, 5, and 10 mg/kg	IL-4↓, IL-5↓ and IL-13↓; IgE production↓	Liu et al. (2013)
Anti-viral activity	*Taraxacum mongolicum*	HepG2.2.15 cells	24 μg/ml	Percentages of HBV-DNA↓; extracellular HBV DNA↓, HBsAg↓, and HBeAg↓ PTBP1↓, and SIRT1↓	Yang et al. (2020)

a Note: ↑, increase; ↓, decrease or inhibit.

### Anti-inflammatory activity

Inflammation describes various physiological and pathological processes triggered by noxious stimuli and conditions, such as infection and tissue injury (Medzhitov, 2008). Akihisa et al. demonstrated that the ID_50_ of taraxasterol extracted from the Compositae flowers was 0.3 mg/ear on 12-O-tetradecanoylphorbol-13-acetate- (TPA-) induced inflammation in mice (Akihisa et al., 1996). After 36 h of treatment with taraxasterol at doses of 2.5, 5, and 10 mg/kg, survival rates in LPS-induced endotoxic shock mouse models were up to 30, 40, and 70%, respectively. Moreover, no toxic effects of taraxasterol were observed in mice that received doses as high as 10 mg/kg. In addition, taraxasterol (10 mg/kg per day) significantly reduced levels of inflammatory cytokines, including tumor necrosis factor-α (TNF-α), interferon-γ (IFN-γ), interleukin-1β (IL-1β), and interleukin-6 (IL-6), and significantly reduced serum levels of inflammatory mediators such nitric oxide (NO) and prostaglandin E₂ (PGE₂) (Zhang et al., 2014). Intraperitoneal injection of taraxasterol (10 mg/kg per day) can significantly reduce the expression of pro-inflammatory factors, myeloperoxidase activity, and lung wet/dry ratio in a mouse model of LPS-induced acute lung injury (ALI). Mechanistically, the anti-inflammatory effects of taraxasterol may be due to the inhibition of the NF-κB and MAPK signaling pathways (San et al., 2014). The nuclear factor NF-κB is considered necessary for the expression of pro-inflammatory genes, and the NF-κB pathway is considered a prototypical pro-inflammatory signaling pathway (Lawrence, 2009). Treatment with taraxasterol at doses of 5 and 10 mg/kg significantly reduced the inflammatory response in a liver injury model induced by concanavalin A (Con A) by inhibiting the toll-like receptor-NF-κB signaling axis. In addition, taraxasterol prevented Con A-induced acute hepatic injury *via* the Bax/Bc1-2 anti-apoptotic signaling pathway (Sang et al., 2019). Furthermore, the increased serum alanine aminotransferase (ALT), aspartate aminotransferase (AST), and hepatic malondialdehyde (MDA) levels induced by Con A were significantly reduced by taraxasterol treatment (Sang et al., 2019). The potent anti-inflammatory properties of taraxasterol (orally with 10 mg/kg per day) have also been demonstrated in mice of an acute experimental colitis (AEC) model induced by oral administration of dextran sulfate sodium (DSS) (Chen W. et al., 2019). Peroxisome proliferator-activated receptor *γ* (PPARγ) plays a central role in the regulation of inflammatory signaling pathways by acting on kinases and transcription factors, such as NF-κB, c-Jun, c-Fos, and nuclear factor of activated T cell (NFAT), and by inhibiting the production of IL-1β and TNF-α (Su et al., 1999; Yang et al., 2000; Desreumaux et al., 2001). In the DSS-induced AEC animal models, taraxasterol (10 mg/kg) reversed DSS-induced PPARγ downregulation in AEC colon tissues and improved DSS-induced colitis, offering a novel insight into potential therapeutic strategies for acute colitis (Chen W. et al., 2019). In addition, Che et al. reported that taraxasterol significantly reduced the expression levels of IL-6 and TNF-α in a dose-dependent manner at doses between 2.5 and 10 μg/ml *in vitro* and 25 and 100 mg/kg *in vivo* (Che et al., 2019). Although there is evidence that taraxasterol has anti-inflammatory effects in various diseases, the precise mechanism by which it regulates inflammatory responses is still unclear. For example, both upstream multiple proteins and some non-encoded RNAs, such as RACK1 (Yao et al., 2014), tripartite motif-containing proteins (TRIMs) (Roy and Singh, 2021), microRNA-144 (Yang et al., 2022), and AMPK (Zhai et al., 2018), can regulate NF-κB expression. It is unclear whether taraxasterol inhibits NF-κB expression by regulating the expression of these proteins. Thus, further discussion is required *in vitro* and *in vivo*.

Recent studies have shown that taraxasterol exerts an anti-arthritic effect. These studies showed that taraxasterol reduced IL-1β-stimulated inflammatory responses *in vitro* and *in vivo* by suppressing the expression of COX-2 and iNOS and reducing NF-κB activation (Piao et al., 2015; Chen J. et al., 2019). Chen et al. reported that taraxasterol suppressed the NOD-like receptor protein 3 (NLRP3) inflammasome through inhibition of the expression of NLRP3, apoptosis-associated speck-like protein containing (ASC), and caspase-1 within a dose range of 0.3 to 0 μm in HFLS-RA cells and with 10 mg/kg in collagen-induced arthritis (CIA) mice (Chen J. et al., 2019). In another investigation, Wang et al. studied the protective effect of taraxasterol against Freund’s complete adjuvant- (FCA-) induced arthritis in rats. They found that taraxasterol (at doses of 2, 4, and 8 mg/kg) inhibited bone destruction by increasing serum OPG production and inhibiting the overproduction of serum inflammatory cytokines (Wang et al., 2016). In addition, Liu et al. showed that taraxasterol (10 mg/kg) improved propionibacterium acnes-induced inflammatory responses in a mouse ear edema model and suppressed pro-inflammatory chemokine production *via* the TGF-β/Smad pathway (Liu et al., 2020). The liver X receptors (LXRs) are members of the nuclear hormone receptor superfamily that bind and are activated by oxysterols (Lehmann et al., 1997). Liu et al. showed that taraxasterol (0 to 12 μg/ml) was a ligand of LXRα and inhibited the expression of TNF-α and IL-1ß *via* the activation of LXRα in LPS-stimulated BV_2_ microglia (Liu et al., 2018). These results suggested that taraxasterol may exert anti-inflammatory effects *via* activation of LXRα in the central nervous system. Similarly, another study on LPS-stimulated human umbilical vein endothelial cells also showed that taraxasterol (5–15 μg/ml) exerted anti-inflammatory effects by activating LXRα (Zheng et al., 2018).

### Anti-oxidative activity

Oxidative stress is caused by exposure to reactive oxygen intermediates, which can damage proteins, nucleic acids, lipids, and cell membranes (Storz and Imlay, 1999). Studies have shown that cumulative damage caused by reactive oxygen species (ROS) contributes to numerous diseases (Apel and Hirt, 2004; Jakubczyk et al., 2020). Several studies have characterized the anti-oxidative effects of taraxasterol. In mice with acute kidney injury (AKI) induced by ischemia/reperfusion injury (IRI), Li et al. showed that taraxasterol (5 and 10 mg/kg) inhibited mitochondrial ROS production and ameliorated apoptosis in the kidney by decreasing Bax expression and increasing Bcl2 expression (Li C. et al., 2020). The transcription factor Nrf2 regulates the expression of phase II detoxification enzymes and a series of antioxidant enzymes (Chen et al., 2015). Heme oxygenase (HO-1), a phase II detoxification enzyme regulated by Nrf2, also plays an important antioxidant role (Suh et al., 2006). Many studies have shown that taraxasterol (2.5–10 mg/kg) inhibited oxidative stress by increasing the activity of the CYP2E1/Nrf2/HO-1 pathway in animal models of ethanol and high-fat diet-induced liver injury (Xu et al., 2018; Li Z. et al., 2020). Moreover, taraxasterol (2.5–10 mg/kg) also reduced the production of ROS, malondialdehyde (MDA), and increased glutathione (GSH) levels and superoxide dismutase (SOD) activity in ethanol-induced liver injury (Xu et al., 2018). An *in vivo* study showed that taraxasterol inhibited cigarette smoke-induced lung inflammation by inhibiting ROS production and ROS-mediated recruitment of TLR4 into lipid rafts within a dose range of 2.5–10 mg/kg. Moreover, taraxasterol also upregulated GSH production (Xueshibojie et al., 2016). In addition, taraxasterol has been shown to exert protective effects against neurological diseases. In oxygen-glucose deprivation/reperfusion- (OGD/R-) induced hippocampal neurons, taraxasterol (2.5–10 μm) significantly suppressed ROS production and MDA generation. Furthermore, taraxasterol induced nuclear Nrf2 accumulation and promoted increased expression of HO-1, NQO-1, and GPx-3 (He et al., 2020). Wang et al. found that taraxasterol increased the phosphorylation level of ERK1/2 in a cardiomyocyte ischemia/reperfusion (I/R) model, which indicated that taraxasterol exerted protective effects against oxidative stress by upregulating the ERK pathway at a dose of 30 μm (Wang et al., 2018). These results demonstrated that taraxasterol protected cardiomyocytes against hypoxia, suggesting that it may be significant for treating heart diseases (Wang et al., 2018).

### Anti-carcinogenic activity

Previous studies have shown that extracts of *Taraxacum officinale* inhibited proliferation and induced apoptosis in hepatocellular carcinoma (HCC), HepG2, and Huh7 cells (Koo et al., 2004; Guo J. B. et al., 2015; Yoon et al., 2016). It has also been demonstrated that taraxasterol inhibits the development and progression of tumors. Taraxasterol extracted from the aerial parts of the *Chrysanthemum* genus significantly inhibited cell proliferation of both PC3 (human prostate cancer) and HT-29 (human colon cancer) cells. The IC_50_ values of the taraxasterol compound were determined as 37.1 and 89.7 µm in the PC3 and HT-29 cells at 48 h, respectively (Boutaghane et al., 2013). However, among other tumor cells, including MCF-7 (human breast carcinoma), HeLa (human cervix carcinoma), SK-MEL-5 (human melanoma), KB (human nasopharyngeal carcinoma), P388 (murine leukemia), MOLT-4 (human acute lymphoblastic leukemia), and SK-OV-3 (human ovary carcinoma) cells, taraxasterol did not exhibit significant inhibitory activity with IC_50_ values equal to or higher than 49 mm, suggesting that the anti-carcinogenic activity of taraxasterol may have cellular specificity (Villarreal et al., 1994; Lee et al., 2010; Jassbi et al., 2016). In an *in vivo* two-stage test, administration with 2 μmol/mouse of taraxasterol markedly inhibited the tumor-promoting effect of TPA on skin tumor formation following initiation with 7,12-dimethylbenz[α]anthracene. In this study, taraxasterol caused an 86% reduction in the average number of tumors per mouse at week 20 (Yasukawa et al., 1996). However, a subsequent study showed that taraxasterol (850 nmol/ml) also exhibited about 60% inhibition of the average number of papillomas per mouse at 20 weeks in terms of the two-stage carcinogenesis test (Takasaki et al., 1999). Furthermore, in the C3H/OuJ female mice treated with taraxasterol (2.5 mg in 100 ml of drinking water), the survival ratio of the mice was 80% even at 70 weeks of breeding, suggesting that taraxasterol can remarkably suppress the spontaneous mammary carcinogenesis in the C3H/OuJ female mice (Takasaki et al., 1999). Histidine triad nucleotide-binding protein 1 (Hint1) is a tumor suppressor often downregulated in association with the development of cancer (Wang et al., 2007; Wang et al., 2009). Bao et al. found that taraxasterol (IC_50_: 17.0 μm) selectively inhibited the proliferation of HepG2 cells by inducing cell cycle arrest at G0/G1 and inhibited apoptosis by upregulating Hint1 transcription to regulate the expression of Bax, Bcl2, and cyclin D1 (Bao et al., 2018). In addition, oral administration of 25 μg/ml of taraxasterol in drinking water for 30 days can effectively inhibit the growth of the implanted SK-Hep1 tumor *in vivo* (Bao et al., 2018). In a gastric cancer subcutaneous xenograft model, taraxasterol (25 μg/ml) inhibited the growth of xenograft tumors by inhibiting EGFR/AKT1 signaling (Chen et al., 2020). The E3 ubiquitin ligase RNF31 is overexpressed in many tumors and is associated with tumorigenesis (Guo J. et al., 2015; Zhu et al., 2016; Qiu et al., 2018). Tang et al. found that taraxasterol (50 μg/ml) promoted the degradation of RNF31 by activating autophagy, thereby inhibiting the p53 degradation and colorectal cancer (CRC) cell proliferation (Tang et al., 2021). In addition, taraxasterol can inhibit cell growth of breast, cervical, and melanoma *in vitro* (Dai et al., 2001; Lee et al., 2010). These findings indicated that taraxasterol may be a promising candidate for treating tumors.

### Others

Liu et al. found that taraxasterol (5 and 10 mg/kg) significantly decreased the production of the Th2 cytokines IL-4, IL-5, and IL-13 in bronchoalveolar lavage fluid (BALF) and reduced the levels of ovalbumin- (OVA-) specific IgE in serum (Liu et al., 2013). In addition, taraxasterol (2.5–10 mg/kg) suppressed airway hyperresponsiveness (AHR) in a dose-dependent manner. Histological studies showed that taraxasterol substantially suppressed OVA-induced inflammatory cell infiltration into lung tissues and goblet cell hyperplasia in airways, which suggested that taraxasterol may protect against allergic asthma (Liu et al., 2013). In an *in vitro* study, taraxasterol (7.5 and 12.5 μg/ml) reduced the number of CaOx crystals in a dose-dependent manner and reduced the diameter of CaC_2_O_4_ dihydrate crystals. In addition, the inhibition of nucleation was increased by taraxasterol in the range of 26%–64% (Yousefi Ghale-Salimi et al., 2018). A recent study found that taraxasterol also exerted anti-viral effects. Taraxasterol (24 μg/ml) significantly reduced the secretion of HBsAg, HBeAg, HBV DNA, and intracellular HBsAg. Moreover, the treatment of taraxasterol (24 μg/ml for 48 h) also decreased the protein expression levels of the host factors polypyrimidine tract binding protein 1 (PTBP1) and sirtuin 1 (SIRT1) in HepG2.2.15 cells (Yang et al., 2020). In addition, pretreatment with different concentrations of taraxasterol (30, 60, 90, 120, 150, 180, or 210 µm) markedly prevented cell injury and inflammation in H_2_O_2_-induced HUVECs by reducing the expression of vascular cell adhesion molecule 1 (VCAM-1) and the cluster of differentiation 80 (CD80) (Yang et al., 2015). In addition, taraxasterol has also been shown to exert significantly antimicrobial effects. Taraxasterol obtained from the Mexican plants of the Asteraceae elicited a significant minimum inhibitory concentration (MIC) value (12.5 μg/ml) against *Staphylococcus aureus* (Villarreal et al., 1994). However, Boutaghane et al. observed weak inhibition of taraxasterol against the growth of Gram-negative (*E. coli*, *P. aeruginosa*, *K. pneumoniae*) bacteria (Boutaghane et al., 2013). Akihisa group described the anti-tubercular activity of taraxasterol against *Mycobacterium tuberculosis* with the MIC values of 64 μg/ml (Akihisa et al., 2005).

## Conclusion

Taraxasterol is a natural pentacyclic triterpene primarily extracted from dandelion. This article gives a general overview of the pharmacological activities of taraxasterol for treating various illnesses, such as respiratory, gastrointestinal, and urinary disorders. Taraxasterol was discovered to have excellent potential for preventing the above disorders. Anti-inflammatory, anti-oxidative, and anti-carcinogenic mechanisms may be responsible for its protective effects.

Neurodegenerative diseases, including Alzheimer’s disease (AD), Parkinson’s disease (PD), and Huntington’s disease (HD), cause progressive damage to the nervous system. The abnormal aggregation of some proteins in particular brain regions is a pathological hallmark of neurodegenerative diseases mainly caused by impaired protein-degradation systems, such as the autophagy-lysosome pathway (ALP) and the ubiquitin-proteasome system (UPS) (Zheng et al., 2014; Finkbeiner, 2020). Recently, it was found that taraxasterol promoted the degradation of RNF31 protein by enhancing autophagy and further alleviating the degradation of p53 through proteasome, indicating that taraxasterol may regulate the protein degradation pathways in cells (Tang et al., 2021). It is necessary to conduct more *in vivo* and *in vitro* studies to determine whether taraxasterol prompts the degradation of aggregate proteins by regulating ALP and UPS pathways in neurodegenerative diseases. Previous studies have shown that inflammatory stimulation can be active by genetic mutation and protein aggregation in neurodegenerative diseases (Glass et al., 2010; Stephenson et al., 2018). Microglia and astrocytes are mainly responsible for persistent inflammatory responses (Xu et al., 2016; Stephenson et al., 2018). Taraxasterol may have effects on inhibiting the inflammatory response induced by glial cells and potentially protective effect on neuronal death caused by abnormal activation of glial cells in neurodegenerative diseases, according to recent research conducted *in vitro*. Taraxasterol inhibited the expression of proinflammatory factors *via* the activation of LXR in LPS-stimulated BV2 microglia (Liu et al., 2018). However, taraxasterol can directly act on glial cells through the blood–brain barrier (BBB), and its concentration in the cerebrospinal fluid requires further investigation.

Additional research is also necessary to identify the effective concentration of taraxasterol in plasma. Zhang et al. reported that the amount of taraxasterol in the plasma of rats following oral administration could be accurately detected through a highly selective and sensitive liquid chromatography/tandem mass spectrometry (Zhang et al., 2015). This finding may aid in the pharmacokinetic study of taraxasterol in humans and other animals. Furthermore, more clinical studies are necessary on the metabolism, bioavailability, and safety of taraxasterol to support its applications in pharmaceuticals and medicine.
